# Next-generation nephrology: part 1—an aid for genetic and genomic testing in pediatric nephrology

**DOI:** 10.1007/s00467-025-06697-2

**Published:** 2025-02-13

**Authors:** Asheeta Gupta, Kushani Jayasinghe, Amar Majmundar, Nina Mann, Rajiv Sinha, Matthew G. Sampson, Catherine Quinlan

**Affiliations:** 1 Dept of Pediatric Nephrology, Great Ormond Street Hospital for Children, NHS Foundation Trust, London, UK; 2 Dept of Paediatric Nephrology, Birmingham Children’s Hospital, Birmingham Women’s and Children’s NHS Foundation Trust, Birmingham, UK; 3 Dept of Paediatric Nephrology, Royal Children’s Hospital, Melbourne, Australia; 4 Kidney Regeneration, Murdoch Research Institute, Melbourne, Australia; 5 University of Bristol, Bristol, UK; 6 Dept of Nephrology, Monash Medical Centre, Melbourne, Australia; 7 Monash University, Melbourne, Australia; 8 Melbourne Health, Melbourne, Australia; 9 Division of Pediatric Nephrology, Boston Children’s Hospital, Boston, MA USA; 10 Harvard Medical School, Boston, MA USA; 11 Institute of Child Health, Kolkata, India; 12 Brigham and Women’s Hospital Kidney Disease Initiative, Broad Institute, Cambridge, MA USA; 13 Dept of Paediatrics, School of Medicine, University of Melbourne, Melbourne, Australia

**Keywords:** Genetics, Genomics, Chronic kidney disease, Utility

## Abstract

Genetic and genomic testing has transformed the practice of clinical nephrology. Yet nephrologists have identified genetic literacy and access to kidney genetic care models as critical barriers to utilising this technology in their practice. To accelerate the implementation of genomic medicine in clinical nephrology, we provide a two-part evidence-based educational review. This inaugural segment highlights the importance of genomic testing for kidney disease and the steps a nephrologist can take to understand and apply this technology to one’s own clinical practice. The second part delineates approaches to operationalising a kidney genetics service based on existing models, reported barriers, and the authors’ experiences in developing kidney clinic models for different settings.

**Graphical Abstract:**

**Figure:**
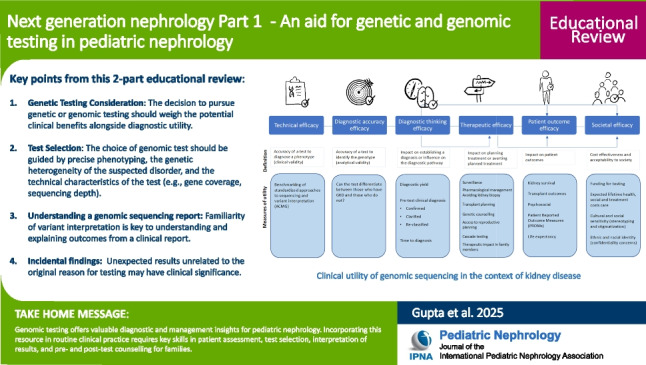
A higher resolution version of the Graphical abstract is available as Supplementary information

**Supplementary Information:**

The online version contains supplementary material available at 10.1007/s00467-025-06697-2.

## Introduction

An estimated 9% of the world’s population, equating to approximately 700 million cases worldwide, is affected by chronic kidney disease (CKD) [1]. Making timely, precise diagnoses that facilitate early kidney protective therapies is essential to reducing the impact of CKD [2]. Broad genetic testing strategies have demonstrated strong utility in the diagnosis of monogenic kidney disease and in enabling precise clinical management for patients and their families [3–6]. Moreover, these testing approaches have demonstrated cost-effectiveness for certain subtypes of kidney disease [7], justifying their use at the *beginning* of a diagnostic workup rather than the end. Despite the potential value to clinical care, integrating this technology into existing practice poses a significant challenge [8, 9].

Genomic medicine services are being introduced across many parts of the world. Technological advances are enabling changes in the way we undertake sequencing with increasing use of broader approaches. There also appears to be a change in terminology to reflect this. The term ‘genetics’ has been defined as the study of genes and their role in inheritance, namely, the way that certain traits or conditions are passed down from one generation to another [10]. In contrast, genomics is the study of all of a person’s genes (the genome), including interactions of those genes with each other and with the person’s environment to affect human health [11]. The World Health Organization has made a distinction between genetic and genomic approaches to sequencing, genetic sequencing being defined as that which scrutinises the function and composition of the single gene, whereas genomic sequencing being that which analyses all genes and their interrelationships to identify their combined influence on the growth and development of the organism [12]. The UK Genomic Education programme echoes this distinction between the terms and adds that genomics encompasses analysis of coding and non-coding parts of the genome [13]. In some settings, such as the UK or Australia, the diagnostic strategies which involve sequencing groups of genes, exons or whole genomes for monogenic disease are referred to as ‘genomic tests’ [14–16]. Though we appreciate that this is not universal, we have used the same term, genomic testing, referring to the sequencing of multiple genes or significant parts of the genome. Using this term to describe a broad approach to genomic analysis helps to highlight the newer methods and concepts underpinning this technology including the risks and benefits nephrologists need to be aware of when using this in their practice. We focus on clinical testing for diseases with Mendelian inheritance. Such conditions have been diagnosed using genomic sequencing in 30–47% of children with CKD [3–5].

The intention of this two-part educational review is to (i) outline why genomic testing should be incorporated into the pediatric nephrology diagnostic toolbox with practical guidance of how this may be done [8, 9] and (ii) summarise current literature and experience of the authors in establishing dedicated kidney genetics clinics in the US, UK, India, and Australia. We will address current challenges in service development and how they may be overcome.

## The value of genomic testing

**Fig. 1:**
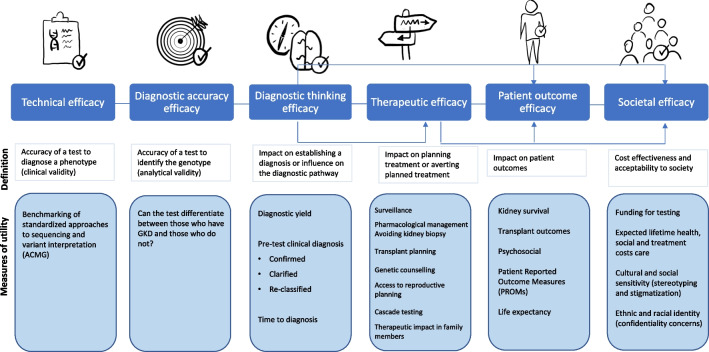
Clinical utility chain of evidence adapted to the setting of kidney disease. Example measures of utility are assigned to clinical utility-related efficacy domains to demonstrate how they can be operationalised and monitored. The model is hierarchical, but the blue lines between domains represent the notion that achieving a given level of efficacy is not always contingent upon the documentation of efficacy at the preceding level (adapted from Hayeems et al. [18], licensed under the Creative Commons Attribution 4.0 International License, https://creativecommons.org/licenses/by/4.0/)

Understanding the benefit or clinical utility of genomic testing for individual patients and their families is important. This utility is wide-ranging and extends beyond obtaining a diagnosis to include impact on therapeutic management, prognostication, patient understanding, and psychological consequences. It also refers to the effect of genomic results on entire healthcare systems, including the economic consequences [17]. Demonstrating clinical utility is part of the rationale for undertaking the test and establishing the ‘usefulness’ of genomic sequencing in kidney disease. This is crucial for its uptake and integration within healthcare systems, government or insurance renumeration, and acceptance by society. One validated model of measuring the clinical utility of genomic sequencing is summarised in Fig. 1 with adaptation to the setting of kidney disease.

### Diagnostic utility

**Fig. 2:**
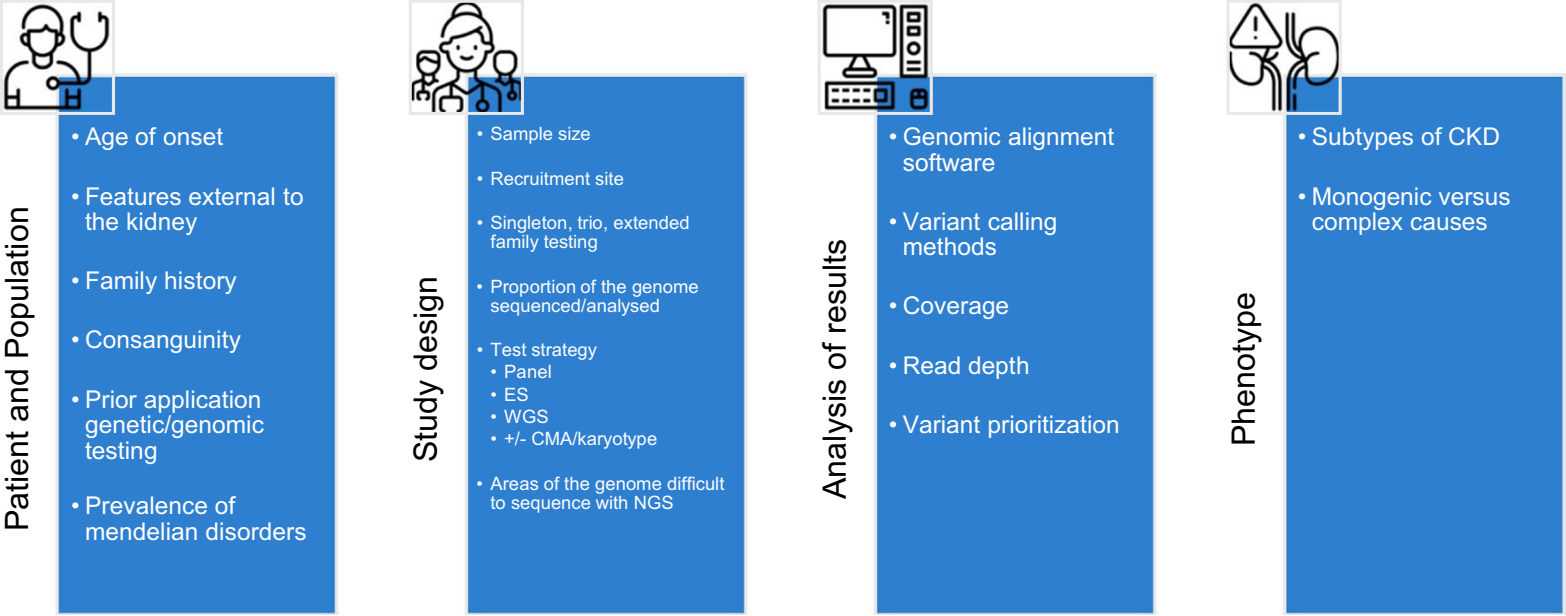
Factors impacting the diagnostic yield of genomic testing [3, 6, 19–21]. Whole-exome sequencing (ES), whole-genome sequencing (WGS), microarray (CMA). Patient factors such as childhood onset of disease, presence of features in organs other than the kidneys, parental consanguinity, and positive family history of kidney disease can increase the diagnostic yield (DY). Prior application of genetic or genomic testing will inform decisions about future testing strategy to include areas of the genome not covered. Population factors such as overall prevalence of consanguinity or Mendelian disorders also impact diagnostic rates. The study design, specifically, larger sample sizes, recruitment from tertiary or specialised centres, trio or extended family testing, and broader sequencing with tailoring to ensure areas of the genome that are not amenable to NGS are analysed in other ways increases the DY. Bioinformatic analysis such as alignment software used, variant calling methods, coverage, and read depth of sequencing as well as variant prioritisation all require careful consideration and can affect the DY. Monogenic conditions and specific subtypes of CKD such as cystic kidney disease, glomerular disease, and tubular conditions have the highest reported DY

Quantifying the impact of genomic testing on rates of diagnoses (diagnostic yield) depends on a range of factors, including the testing strategy and population tested (Fig. 2). Groopman et al. showcased the ability of whole-exome sequencing (ES), with targeted analysis of 625 genes associated with nephropathy, to obtain molecular diagnoses in 9.3% of a predominantly adult, unselected cohort of 3315 participants with CKD [6]. Jayasinghe et al. employed a similar methodology, this time paired with microarray analyses, in a cohort of adult and pediatric patients selected for suspected genetic disease, which demonstrated diagnostic yields of 34% in adults and 47% in children [3]. These patients were prioritised based on presenting features suggestive of a monogenic cause, the presence of a family history of kidney disease, or childhood onset of an underlying syndrome. Mann et al. demonstrated the diagnostic utility of ES with targeted analysis of 396 genes, in a predominantly pediatric cohort of 104 kidney transplant recipients [4]. A monogenic cause was found in 33%, with definitive diagnoses obtained in 56% of patients with CKD of unknown cause. Some genetic results would have significantly impacted transplant planning if they had been discovered earlier. This finding made the case for earlier application of genomic sequencing before kidney transplantation. The diagnostic yield varies widely between studies that use genomic sequencing for suspected monogenic kidney disease. For this reason, it is important to determine if published results can be applied to one’s own practice. Figure 2 elaborates on factors that impact the diagnostic yield, critically appraising studies of interest.

Genomic testing can add further depth to an a priori clinical diagnosis. Referring to the study by Groopman et al., clinical diagnoses were confirmed by genomic testing in 27% of study participants. This genotype-level knowledge added value by providing additional clinical insight, estimation of the risk of disease progression, guidance for family counselling and donor selection for transplantation [6]. Clinical diagnoses were clarified in 76% of cases by identifying a specific molecular cause within the broader category of clinically suspected disease. An example is discovering pathogenic variants in the *COL4A5* gene as a cause of Alport syndrome. This information provides specific information about mode of inheritance, in this case X-linked, thereby helping select potential at-risk family members for testing. Remarkably, for CKD of unknown origin, where neither clinical phenotype nor non-genomic testing could determine etiology, a molecular diagnosis was found in 23% of cases. Finally, there was complete re-classification or revision of clinical diagnoses in 11%, illustrating the heterogeneity and overlap in phenotypic features across different subtypes of CKD. This was most prominent in diagnoses of Alport syndrome, for which only 38% of all genetically confirmed cases carried this clinical diagnosis. The remainder had been labelled as focal segmental glomerulosclerosis (FSGS), unspecified glomerulopathy, hypertensive nephropathy, or CKD of unknown cause. In contrast, ADPKD had a strong correlation between clinical and molecular diagnosis.

### Clinical utility

The therapeutic benefit of genomic testing is continuously evolving and can be challenging to measure. It has received much less focus than diagnostics [22]. Ideally, all aspects of clinical utility should be considered when deciding whether to pursue genomic testing. Powerful utility data from Jayasinghe et al. (adults and children) [2] and Chen et al. (children) [4] demonstrated the clinical value of ES. These studies supported targeted pharmacological therapy, cessation of unnecessary medications, and accurate surveillance of extra-renal manifestations. Furthermore, kidney biopsy was avoided in up to 35% of cases. Utility data from Dahl et al. using a 385 gene panel for an adult cohort affected with CKD found 90% of those with a positive diagnosis (*n* = 338, 20%) had a result which impacted their management [23]. Beyond the individual patient, relatives of affected individuals can undergo genetic counselling and testing through a systematic process known as *cascade screening*. A positive result can identify at-risk family members even before symptom onset [24]. A negative result in this context means the relative does not have the familial disease caused by the specific genetic change found in the proband. Having a negative result may still have clinical utility, such as stopping surveillance or enabling kidney donation. Utility data has demonstrated that most genetic results influence genetic counselling for patients and families and cascade testing is accessed by 35 to 79% of at-risk relatives [3, 5].

## Who to test

Selecting factors that are independent predictors of a positive test can help to prioritise patients for diagnostic genomic testing. This includes childhood onset, a family history of kidney disease, consanguinity, and extra-renal features [3, 5, 6, 19]. Though these factors appear to be investigated in many studies that evaluate the diagnostic yield of genomic sequencing, they are not consistently found to be independent predictors of a positive genetic result. We presume this is due to variation in composition of the populations studied. Independent predictors of a positive genomic test may not always be evident in every family, or easy to identify, and their absence should not preclude one from proceeding with testing if the results will have clinical use. CKD subtypes, specifically glomerular, tubular, or cystic kidney disease have demonstrated the highest diagnostic yields across adult and pediatric studies [3]. Hence, the most recent yields in patients selected for presence of the features mentioned above approach 80% [25–27]. Though these highly selected cohorts may not reflect the diagnostic findings in more general patient groups, they may be used to prioritise patients for genomic sequencing.

## When to test

Crucial health economic work has confirmed that genomic sequencing is cost-effective, with the greatest gains achieved when performed at the point of referral as part of the initial diagnostic work-up [28]. Benefits include offering targeted treatments, avoiding additional investigations (‘the diagnostic odyssey’), discontinuing futile therapies, offering cascade testing, and improving future parental reproductive outcomes. These observations have led to the development of rapid sequencing pipelines that demonstrate high diagnostic and clinical utility, reducing the cost per diagnosis by making further savings through avoiding unnecessary tests and procedures [29]. In pediatric nephrology, using ES as a first-tier investigation for children with glomerular disease has shown health economic benefit (incremental cost saving of $2 K USD per additional diagnosis) with a superior diagnostic yield (42% using early ES vs. 4% from the standard diagnostic pathway, without genomic sequencing) [7]. Further health economic evaluations have supported this finding [30, 31].

## Phenotyping

Phenotyping is the process of analysing an observable trait, encompassing collation of a medical and family history and existing clinical test results. An accurate phenotype is the bedrock upon which variant assessment is developed. Key medical history data include the age of onset, presenting kidney symptoms, disease trajectory, and physical examination findings including features outside the kidney. Applications such as Face2Gene can assist the nephrologist working without access to clinical genetics support, in defining facial dysmorphologies [32]. However, this cannot replace the key role of a trained clinical geneticist. Examples of kidney diseases with characteristic craniofacial features include the following:

- Sensenbrenner syndrome (caused by pathogenic variants in *IFT140*, *IFT122*, *WDR35*, *IFT140*, *IFT43*, *IFT52*, and *WDR19)* which is associated with sparse, slow-growing fine hair, hypodontia (absence of one of more teeth) and/or microdontia (small teeth), dolicephaly (sagittal suture synostosis), and a range of kidney phenotypes [33].
- Schimke Immuno-osseous dysplasia (caused by pathogenic variants in *SMARCAL1*) can be associated with a wide, depressed nasal bridge and a broad nasal tip, hyperpigmented macules (70%) that can extend to the neck and face though mainly found on the trunk, microdontia, hypodontia, and/or malformed molar teeth, and is associated with steroid-resistant nephrotic syndrome (SRNS) [34].
- Structural ear signs can be associated with syndromes involving kidney disease. Townes-Brocks syndrome (caused by pathogenic variants in *SALL1*) is associated with dysplastic ears (87%) with overfolded superior helices and microtia (small, abnormally formed external ear); branchio-oto-renal syndrome (caused by pathogenic variants in *EYA1*) is associated with pre-auricular pits and auricular malformations [35, 36].

Family history should include a pedigree of affected and unaffected individuals to help determine a suspected inheritance pattern. Where possible, obtaining at least three generations is ideal. Including birth history and disease in other organ systems (which may be associated with suspected genetic disease etiology) is useful for each family member.

Physicians should ensure questions to ascertain family ethnic origins are not missed, as certain ancestral groups are at risk of genetic disease secondary to specific founder variants, as well as potential consanguinity. Founder variants are those that are observed with high frequency in populations that were culturally or geographically isolated, and are inherited from a common ancestor [37]. This process can be augmented by using a questionnaire which can act as an aide-memoire and by consulting with other professionals also involved in providing care to the patient or family. Performing clinical tests to evaluate the primary kidney disease is part of the phenotyping process. This includes any relevant non-genomic investigations (blood tests, urinalysis, imaging, histopathology) as well as review of prior genetic or genomic testing. Clinicians should consider if existing clinical tests point to manifestations of genetic disease: for example, tubular proteinuria in patients with a proximal tubulopathy or liver abnormalities on an abdominal ultrasound from a patient with cystic kidney disease. It is important to note that children who are asymptomatic, but have a family history of genetic disease, which increases their likelihood of developing it, fall under the category of predictive or pre-symptomatic testing. For these cases, referral or consultation with clinical genetics is important to jointly explore the clinical utility of testing and ethical implications and to offer appropriate counselling for the family before proceeding.

## Genomic testing modalities

Only after careful phenotyping should a genomic test be considered. Genomic tests typically involve the use of microarray technology or massively parallel sequencing [38] and include chromosomal microarray (CMA), targeted sequencing (panels), exome sequencing (ES), and whole-genome sequencing (WGS) (Table 1). The phenotype of interest should determine the choice of genomic test. This will be influenced by local availability, funding, and the genomic proficiency of the nephrologist. Key questions to consider at this point are whether the genomic test (i) adequately covers all genes related to the suspected genetic diseases, (ii) covers all the exons in those genes well, and (iii) detects small nucleotide changes as well as larger deletions in those genes. Time, experience, and a good relationship with the local clinical genetics department is key to building the specific knowledge base in this area.

**Table 1:** An overview of genetic tests used in diagnosing kidney related conditions. *Abbreviations*: *ADPKD* autosomal dominant polycystic kidney disease, *aCGH* array comparative genomic hybridisation, *aHUS* atypical haemolytic uraemic syndrome, *bp* base pairs, *CMA* chromosomal microarray, *CNV* copy number variant, *CGP* clinical gene panel, *DY* diagnostic yield, *NGS* next-generation sequencing, *RCAD* renal cysts and diabetes syndrome, *SNP genotyping* single-nucleotide polymorphism genotyping (*note this can be undertaken using array or next-generation/massively parallel sequencing strategies), *VUS* variants of uncertain significance

Test	Applications	Examples of specific uses	Scope	Advantages	Limitations	DY	Reference
Single gene sequencing (Sanger sequencing)	Suspected monogenic conditions/minimal locus heterogeneity Segregation analysis Prenatal diagnosis Confirmation of NGS results Sequencing regions not amenable to NGS (i.e., highly repetitive sequences or homologous regions) Assessment of low-level mosaicism	*CTNS* **(Cystinosis)** * PKD1/2* **(ADPKD)** * WT1* **(Denys-Drash)** * GLA* **(Fabry)**	Sequence variants Small insertions and deletions (< 50 bp)	High analytical accuracy Negligible risk of VUS or incidental findings Low cost Useful for sequencing genes not amenable to NGS	Cost and time implications increase with number and size of regions sequenced	Up to 100%	[22, 39]
Karyotype	Suspected chromosomal abnormality Determine recurrence risk of aneuploidies Consider if associated dysmorphism or multisystem signs/symptoms (especially neurodevelopmental)	Trisomy 1313 **(Patau syndrome)**	Large deletions and duplications Balanced rearrangements Chromosomal rings CNV (4-6Mb)	Low cost Low turn-around-time	Low resolution	3%	[40]
**CNV analyses**							
Chromosomal microarray (CMA) Includes: array Comparative Genomic Hybridisation CGH (aCGH) Single Nucleotide polymorphism (SNP) genotyping*	Aid diagnostics in patients with multisystem congenital anomalies especially intellectual disability, developmental delay, and autism spectrum disorder Suspected CNV	*HNF1B* **(RCAD as part of the17q microdeletion syndrome)** 7q11.23del **(Williams syndrome)** **CAKUT** *NPHP1 ***(Nephronophthisis)**	CNV (up to 25 kb)	Genome-wide analysis Higher resolution than karyotyping Requires no prior knowledge of the chromosome imbalance involved Risk of VUS low using CMA Moderate cost implications Custom-designed oligonucleotide aCGH with greater resolution has enabled increases in DY * Useful in assessing paternity when *de* *novo* variant suspected. This can be undertaken using array or NGS/MPS based sequencing strategies	Cannot identify balanced rearrangements or sequence variants Limited ability to detect small CNV (<10kb) or those in pseudogenes/repetitive regions as well as low level mosaicism Lower risk of VUS compared to NGS strategies Risk of incidental findings	15–20%	[41, 42]
Multiplex Ligation-dependent Probe Amplification (MLPA)	Suspected CNV	*CFH, CFI, CD46, CFHR1, CFHR3 ****(aHUS)***	CNV large (chromosomal) and small (exonic)	Low turn-around-time Low cost	Can be associated with false-positive results, rates of which vary according to clinical condition and probe design		[43, 44]
**Next-generation/massively parallel sequencing strategies**							
Clinical gene panel (CGP)/gene list	Phenotypes pointing to a specific condition with locus heterogeneity Conditions with overlapping phenotypes Conditions with a common pathway	**Steroid resistant nephrotic syndrome** **Hereditary tubulopathies** **Complement-related disorders**	Sequence variants Small insertions/deletions (< 50bp) Larger CNV	Cost, turn-around-time, data storage requirements relatively low compared with other NGS strategies Risk of VUS and incidental findings is relatively low compared with other NGS strategies but increases as panel size increases	Not suitable if there is no primary phenotype or differential diagnosis is too long Panel content is static, requiring regular review and update with newly discovered genes Panel content can vary between laboratories Some genomic regions are poorly covered (i.e., highlyrepetitive sequences (*MUC1)* or homologous regions (*PKD1)*)	Varies according to phenotype under investigation	[39]
Whole-exome sequencing (WES)	Conditions that exhibit phenotypic or genetic heterogeneity Previous testing has been negative or cases where a specific test is unavailable	**Unexplained CKD**	Sequence variants in coding parts of genome, small insertions/deletions (< 50 bp), or larger CNV dependent on the algorithms or computational tools used	More flexible analysis than gene panels, with the opportunity to identify new causative genes and broaden the phenotypic spectrum of existing conditions Data is ‘future-proof’ - Data can be stored for future re-analysis as new genes are discovered and variants re-classified	Intermediate turn-around-time, cost, and data storage requirements (lower cost than WGS) Risk of VUS and incidental findings is higher than gene panel testing Some genomic regions are poorly covered (i.e., highly repetitive sequences (*MUC1)* or homologous regions (*PKD1)*)	Varies greatly depending on the clinical indication and data capture systems used	[18, 45]
Whole-genome sequencing (WGS)	Conditions that exhibit significant phenotypic or genetic heterogeneity Cases where previous testing has been negative or cases where a specific test is unavailable		Sequence variants in the whole genome, small insertions/deletions (< 50 bp) Structural rearrangements including CNV, translocations and inversions	In addition to advantages of WES, WGS can identify deep splice site/intronic variants not detectable using other NGS strategies Able to identify CNV Data is ‘future-proof’ - Data can be stored for future re-analysis as new genes are discovered and variants re-classified	High turn-around-time, cost, and data storage requirements (compared with CGP and WES) The volume of incidental findings and VUS increase in line with the amount of genomic data analysed Not yet routinely used in clinical practice Some genomic regions are poorly covered (i.e., highly repetitive sequences (*MUC1)* or homologous regions (*PKD1)*)	Varies greatly depending on the clinical indication	[21, 39]

Chromosomal microarray (CMA) involves hybridising patient’s DNA to a microchip-based testing platform that uses labels or probes to bind to specific DNA regions [46]. This technique detects smaller sub-microscopic duplications or deletions of chromosomal segments that are too small to be detected by light microscopy using conventional cytogenetics. Microdeletions are usually less than 5 megabases (5 million base pairs) in length and involve several contiguous genes [47]. CMA can detect copy number variants (CNVs). The term CNV refers to the number of copies of a particular section of DNA, which differs between individuals [48]. These CNVs can vary in length, may or may not contain a gene, and have arisen through duplication or deletion events. CMA also detects longer than expected homozygous genomic segments. These are regions where there is inheritance of identical alleles, also known as long continuous segments of homozygosity (LCSH), and can imply risk for recessive disease, imprinting disorders (where a gene from one parent is expressed and the gene from the other parent is silenced) or presence of uniparental disomy (where both copies of a genomic region are inherited from one parent). The most appropriate scenarios for CMA use include cases of kidney disease where a deletion is suspected such as simultaneous deletion of *TSC2* and *PKD1*, given their close proximity on chromosome 16p. Another important example where CMA can be useful is when *HNF1B*-related kidney disease (also known as renal cysts and diabetes syndrome (RCAD)) is suspected. CMA can detect a microdeletion of the 17q region which contains the *HNF1B* gene, leading to RCAD with additional features of autism or intellectual disability.

Targeted sequencing uses defined probes to investigate individual genomic regions of interest, so that only specific genes or coding regions within genes known to commonly harbour disease-specific variants are sequenced. This can be a faster and more economical way to achieve a clinically relevant result. A traditional panel test includes targeted sequencing of a predefined list of genes. Another example is sequencing of just the protein coding regions (exons) in areas of interest, referred to as targeted exome sequencing (TES). Broader testing encompassing analysis of all coding regions is referred to as whole-exome sequencing (ES). ES sequencing data can also undergo targeted analysis of regions of interest and may also be referred to as TES. The broadest approach uses WGS to establish the order of the building blocks of DNA, adenine, cytosine, guanine, and thymine, in the entire genome. This includes protein coding exons and non-coding regions such as introns or intergenic regions. The exome comprises only 2% of the genome and is thought to harbor about 85–90% of all disease-causing variants in rare Mendelian genetic disorders [48]. However, there are emerging reports of variants in intronic and deep intronic regions resulting in kidney disease. An example of this is a case of Alport syndrome caused by aberrant gene splicing due to pathogenic variants in an intronic region [49]. Gene splicing is the process by which introns, the non-coding regions of genes, are excised out of the primary messenger RNA (mRNA) transcript. When gene splicing is altered, the primary mRNA can be truncated (shorter) or contain missing domains which can alter protein function.

## Economic considerations

Re-analysis of sequencing data (after a pre-determined time interval) is one strategy to try and obtain a diagnosis for patients with a negative result and a high suspicion of genetic kidney disease. ES with subsequent re-analysis is currently the cheapest way to secure a molecular diagnosis for a suspected Mendelian condition [50]. One study comparing the diagnostic rate between WGS and ES with re-analysis showed that while 34% (13 of 38 families) received a diagnosis from WGS, if ES data had been re-analysed 2 years later, an estimated 54% (7/13) of these diagnoses would have been identified [50]. The WGS-specific diagnostic rate of 16% (6/38) would have been even lower with use of newer ES techniques, which offer better coverage of diagnostic variants. Therefore, WGS offers a slight diagnostic advantage, but ES with re-analysis identifies most diagnostic variants. Interestingly, economic analyses for ES-negative cases revealed the incremental cost per additional diagnosis was lower using WGS following ES re-analysis when compared with WGS alone to achieve the same diagnostic yield, $23 K, versus $27 K USD. This saving is due to the lower costs associated with ES re-analysis and the need to use WGS in only those where re-analysis of ES is negative. It is important to note that ES re-analysis requires a time interval of 18 months to 2 years to enable sufficient progress in gene disease knowledge and improved bioinformatic analyses including automated genomic pipelines [20, 51, 52]. Exome data obtained prior to 2015 is not comparable to that obtained more recently, due to poor coverage of relevant regions that newer platforms can achieve. Therefore, re-analysis of sequencing data prior to 2015 is not advised. These cases require re-sequencing.

Utilising WGS as a first-line test gives maximal diagnostic yield; however, it is associated with an incremental cost of each additional diagnosis compared to ES of $19 K USD. The ability to choose this diagnostic strategy depends on the availability of WGS and the expertise and technical support required to analyse such data, which is lacking for many centres. Furthermore, due to the larger amounts of data generated with WGS compared to ES, the cost and logistics of long-term data storage are significantly greater. Large-scale genome sequencing projects have drawn attention to implementing IT infrastructures that facilitate centralised and harmonised data processing [53], enabling specialised centres to interpret and analyse data while concurrently maintaining data security and quality checks. Cloud computing has been used in some jurisdictions to enable data sharing with remote centres. It has been proposed that this model of sharing resources through cloud computing is helpful in minimising overall cost, space, and data infrastructure while maintaining security [53]. In summary, using ES with re-analysis is the most cost-effective option, but it requires additional time and up-to-date technology.

## Gene lists and crowdsourcing tools

We currently advocate that the team begin genomic analysis by examining only those genes concordant with their patient’s phenotype. Prespecifying a list of genes, based on the pre-test probability of a child having a specific disease, will increase the test’s positive and negative predictive value [54]. Within a disease category, selecting a gene panel test can be more complicated. Commercial options for the same disease can vary in the number and specific genes analysed. Therefore, nephrologists must ensure the genes of interest are included in the test. Furthermore, gene lists require regular review and updates as new gene-disease associations emerge. Consequently, there has been a move to undertake ES with analysis of just the genes of interest (also termed a virtual panel, or targeted exome sequencing). This strategy offers the potential to analyse sequencing data more broadly or in different ways as new gene-disease associations emerge. This is also important when there is high clinical suspicion of a specific condition despite negative genomic test results.

Fortunately, substantial work has been done to curate clinical phenotype-driven gene lists in pediatric nephrology. These gene lists are compiled through large collaborative activities and represent expert consensus on which genes have sufficient evidence to attribute clinical causation. They will usually contain curated lists that meet guidelines from the American College of Medical Genetics (ACMG) such as PanelApp [55] and the Clinical Genome Resource (ClinGen) [56]. PanelApp is a publicly available, open-source knowledge base for virtual gene panels. The resource combines expert reviews and manual curation to create diagnostic-grade gene lists [55]. Initial gene panels relevant to a rare disease diagnosis or phenotype are created from standardised, pre-defined sources that provide high-quality gene-disease information. They are then given an initial confidence rating indicated by a traffic light system. Guidelines for ascribing evidence are based on those utilised as part of ClinGen, enabling alignment of PanelApp datasets with other gene curation endeavours [55]. Curators can add updates for key journals, ClinGen, and OMIM alerts, adding to the learning opportunity for nephrologists.

The Clinical Genome Resource (ClinGen) is a National Institute of Health-funded resource that aims to create a central, public repository of genes and variants for use in precision medicine and research. Data are compiled through a systematic review of existing standards, evidence frameworks, and biocuration tools that support a global expert curation effort from a wide range of participants. Building on gene lists from Panel App, the ClinGen Gene Curation Expert Panels (GCEPs) have brought together experts from different backgrounds to contribute to curation efforts and the production of rigorously reviewed phenotype-specific gene lists. These groups include clinicians, clinician scientists, researchers, bioinformaticians, scientific curators, and students, encompassing experience from the clinic, academia, and industry. This is particularly helpful in engaging with individuals with expertise in a given rare disease, where there may only be a handful of experts, or a few published cases worldwide and includes adding to gene lists from other diagnostic laboratories or research groups. In this way, expertise and knowledge accessed through crowdsourcing in PanelApp can help to establish a final set of phenotype-specific genes with a high level of evidence for use in genomic analysis through ClinGen. Alongside this, the Gene Curation Coalition (GenCC) is working to develop universal standards for defining genotype and phenotype evidence [53]. Additionally, standards produced by the GenCC, PanelApp, and GA4GH (Global Alliance for Genomics and Health) members are being formulated to further support global sharing of gene-disease curation outputs.

The diagnostic utility of PanelApp gene lists have been evaluated for a cohort of probands with rare disease in the UK. Use of a curated gene list from PanelApp either as a single method or in combination with phenotype-driven gene lists (for patients with more than two systems affected) was applied to ES data from a cohort of children with abnormalities of the neurological system including intellectual disability (54%), head and neck (19%), skeletal system (16%), ear (15%), and eye (15%). A significant reduction in variant interpretive workload was achieved by using the phenotype-driven virtual panels compared with standard strategies of variant filtering. An overall diagnostic yield of 24% (96/400) was achieved using ES and application of a virtual panel. This was deemed to be comparable to ES without targeted analysis [57]. In this way, expertise and knowledge accessed through crowdsourcing in PanelApp can help to establish a final set of phenotype-specific, evidence-based genes for use in genomic analysis through ClinGen.

## Interpretation of results—variant curation

The next challenge lies in interpreting the results and applying them to clinical care. The academic or commercial diagnostic laboratory curates variants identified through sequencing per guidelines from the ACMG and the Association for Molecular Pathology that were produced in 2015 [17]. These criteria help to standardise variant assessment in the clinical setting and were produced in response to a perceived abundance of tenuous genotype–phenotype associations in the literature [58]. The guidelines ensure robust evidence for variants is collated systematically to make a disease assertion. Application of these guidelines is restricted to inherited Mendelian conditions with high penetrance (likelihood of manifesting signs or symptoms of the genetic disorder) and caution should be used in their application to pharmacogenomics, complex traits, or secondary findings [59]. Variant classification involves interpreting information about the specific variant. To classify a variant’s pathogenicity, a range of resources are utilised to gather population, conservational, computational, functional, family segregation, and allelic data [17].

### Frequency of an allele in the population

Evaluating the allele frequency (AF) of a pathogenic variant in a reference population allows us to determine if it is rare enough to be disease-causing. Variation at a single base position is referred to as single-nucleotide polymorphism (SNP) [48]. At a given SNP, the most common allele is referred to as the major allele, whereas the least common (or rarer) allele is called the minor allele [60]. Alleles are variant forms of DNA sequences that are located at the same position, or genetic locus, on a chromosome. The AF expresses how often the allele of interest is observed in a population divided by the total number of copies of all alleles at that particular genetic locus in the population [48]. The minor allele frequency refers to the rate at which the second most common allele (minor allele) occurs within a particular population for a specific genetic locus or variant [61]. The minor allele frequencies (MAF) reflect the relative amounts of genetic diversity in populations at a given locus. The MAF is usually expressed between 0 and 0.5 (since, by definition, the minor allele cannot be more frequent than the major allele). A variant that causes a rare disease can be defined as one with a MAF of 0.01 or less for a recessive condition [62]. Minor allele frequencies more than 0.01 are typically considered to reflect benign variation within a general population. The ACMG guidelines use a MAF of more than 0.5 to define a variant as being benign [63].

Freely accessible catalogues of sequencing data from worldwide cohorts make this evaluation of population-level AF possible. One example is the Genome Aggregation Database (gnomAD) [61]. GnomAD version 4 contains ES sequencing data from 730,947 individuals and WGS data from over 76,215 individuals. This resource has been used in ACMG guidelines. Notably, the genomic data in gnomAD were primarily obtained from case–control studies of adult-onset disease [64] and therefore are not ‘healthy control’ cohorts. For example, ~ 1/1000 individuals in the gnomAD cohort have pathogenic variants in genes associated with ADPKD [65], suggesting that unrecognised cases of ADPKD may be present in this cohort. It also does not represent all populations equally, so caution should be applied when analysing a patient’s data if they are from an under-represented ethnic minority. However, rare and severe genetic conditions with onset of symptoms during infancy or childhood are unlikely to be represented in this database making this resource invaluable for variant interpretation for such diseases.

### Disease-related genetic variation databases

Disease databases such as ClinVar [65] or the Human Genome Mutation Database (HGMD) [66] inform whether a variant is known to be associated with disease. The ClinVar database documents genomic variants and phenotypes submitted by researchers, clinicians, and laboratories, providing a valuable resource which gives strong evidence for pathogenicity when the variant under investigation is noted to be present in an individual with a similar phenotype. With time, the number and depth of population databases of healthy individuals will increase as researchers seek to develop genotyped cohorts of healthy elderly people, such as ASPREE-G [67]. This cohort consisted of 16,703 Australian and 2411 US participants with a median age of 74 (range 65 to 98 years), 56% women. Minority ethnic groups made up 9% of the total cohort, and 55% of the US cohort. Rates of hypertension, obesity, and CKD were like age-matched populations from both countries. Efforts must be made to ensure there is ethnic diversity within databases to ensure equitable application and interpretation of genomic testing across all populations. Several projects are underway to do just this, including Our Future Health (UK), All of Us Research Program (USA), OurDNA (Australia), Mexican Biobank (Mexico), and H3Africa (sub-Saharan Africa) [68].

### Segregation and allelic data

Demonstrating that a candidate variant segregates with disease in the family increases the likelihood that it may be a disease-causing variant. Co-segregation analysis involves assessing if the genotype under evaluation is found in other family members. This includes testing first degree relatives that share the same disease phenotype and those that do not [48]. Measuring how often an allele and a disease are inherited together in a pedigree is a component of the ACMG approach for assessing germline variant pathogenicity [17]. Ideally, testing as many first-degree relatives as possible increases the amount of informative meioses and provides a more comprehensive view of the inheritance pattern within families. This improves the power, accuracy, and validity of segregation analysis [69]. Co-segregation analysis can determine the pathogenicity of a homozygous variant by demonstrating that both unaffected parents are heterozygous carriers. It can be of further use in determining the pathogenicity of compound heterozygous or autosomal dominant variants. For instance, parental testing can determine whether compound heterozygous variants are inherited in cis phase (e.g. inherited from the same parent and located on the same chromosome) or in trans phase (e.g. inherited from different parents and located on different chromosomes). It can also be utilised to establish de novo inheritance, in which a mutation arises during gamete production, fertilisation, or embryo development in the child. De novo inheritance is more commonly seen in autosomal dominant conditions, in which both parents are unaffected. This comes with the caveat that paternity should be confirmed and in certain circumstances, such as assisted reproduction, maternity should also be confirmed.

For autosomal recessive diseases, pathogenic alleles in trans phase (e.g. one from each parent) are supportive of pathogenicity. The ability to perform co-segregation analysis by sequencing of parents thus increases the diagnostic rate. It is easier to achieve in pediatric practice than in adult nephrology where older patients have a much lower probability of having parents available for testing because of geography or death.

### Computational assessment of conservation and modelling for functional prediction

Bioinformatic or in silico tools, based on computer modelling, can predict pathogenicity and depend on diverse types of information, such as sequence conservation or modifications at the protein level.

In silico tools that assess conservation data are based on the expectation that the likelihood of a variant being truly pathogenic is increased if this results in a change of amino acid residue in a highly conserved position across animal species. This would suggest that an amino acid was preserved in the face of selection pressures during evolution (from single-cell eukaryotes to higher level mammals). Variants predicted to cause loss-of-function are mostly expected to be deleterious. This includes nonsense (alterations that cause the premature termination of a protein) or frameshift (an insertion or deletion that disrupts the triplet reading frame of a DNA sequence) variants. In silico tools are especially helpful for evaluating missense variants (single base pair substitutions that alter the genetic code resulting in a change of amino acid) [48]. This is because not all missense variants will alter protein function though they alter the amino acid. As variant classification is inaccurate and inter-tool agreement is poor, multiple in silico tools are generally used to reach consensus regarding pathogenicity [70]. Different laboratories use different tools in different combinations, with a general approach of requiring two or more to judge a variant as harmful and support pathogenicity. Analogous in silico approaches should be employed for splice site-associated variants (variants occurring at the boundary of an exon and intron) to predict the likelihood of such variants causing impaired splicing. While canonical splice site changes can be more readily interpreted with these tools, extended splice site changes may ultimately warrant evaluation by messenger RNA transcriptional studies in patient samples.

Lastly, the availability of protein structural data from experimental data (e.g. X-ray crystallography, NMR spectroscopy, cryo-electron microscopy) has increased by tenfold over the past 2 decades (see rcsb.org). Computed structure models, including alpha-fold, have led to a further explosion of structural biology information in the past several years [71, 72]. These models can provide insights not obtainable from primary amino acid sequences by demonstrating three-dimensional relationships between a variant-associated amino acid and distant protein parts. This can reveal critical biological knowledge for variant interpretation. Tools to predict the impact of variants on structural stability are available but less reliable than experimental methods, so should be employed only in combination with other computational or biological approaches to investigate a genetic variant [73]. Overall, in silico approaches can evaluate a genetic variant at multiple levels. A summary of some of the tools and scores used in variant interpretation are given in Table 2. Clinicians must seek genetics or biology expertise as needed to employ these tools.

**Table 2:** Examples of in silico (computer-based modelling) tools and methods of scoring to aid variant interpretation

**Tool**	Role	Further information
SIFT (Sorting Intolerant From Tolerant)	Predicts whether an amino acid substitution will affect protein function based on sequence homology	https://sift.bii.a-star.edu.sg
MutationTaster	Integrates evolutionary conservation data, splice site prediction, protein features, and mRNA stability data to evaluate the impact of a single nucleotide variant	https://www.mutationtaster.org
CADD (Combined Annotation Dependent Depletion)	Integrates conservation and functional information into one metric	https://cadd.gs.washington.edu
Polyphen 2	Predicts the impact of an amino acid substitution on the structure and function of the protein	http://genetics.bwh.harvard.edu/pph2/
Mutation Assessor	Based on evolutionary conservation of affected amino acid in protein homologs	http://mutationassessor.org/v1
**Method of scoring**		
Grantham score	Predicts the effect of substitutions between amino acids based on chemical properties including polarity and molecular volume	Further details and information on how these and other methods of scoring may be used in practice can be sought from the Association for Clinical Genomic Science [70]
REVEL score (Rare Exome Variant Ensemble Learner)	Indicates the likelihood of a missense variant being disease causing based on a combination of predictions from a range of tools	

### Functional data

Functional assays can be used to assess the variant’s in vitro and in vivo impact. As with in silico modelling, functional approaches warrant strong collaboration with biologists, as variants’ pathophysiologic impact can have complex implications for cell biology and tissue homeostasis. In some instances, biological functions of a disease have been well studied and the impact of variants documented. For instance, previous reports measuring alpha-galactosidase activity associated with Fabry disease-associated *GLA* variants provided vital data to distinguishing pathogenic from non-pathogenic variants in *GLA* [74]. However, these types of functional studies are not always uniformly performed in research laboratories for variants after the initial discovery of a novel disease gene.

### Reverse phenotyping

A key step in variant interpretation is reverse phenotyping, where the primary nephrologist determines if their patient has clinical manifestations beyond the initial kidney phenotype linked with the variant-associated genetic condition. These manifestations can be queried in the literature but also resources like OMIM (Online Mendelian Inheritance in Man). Such phenotyping should certainly begin as a non-invasive approach by obtaining further history from the patient and querying their medical record. New clinical testing should only be considered in cases associated with (i) a clearly pathogenic or likely pathogenic variant (see below), (ii) where further clinical testing would be indicated based on the genetic diagnosis, and (iii) after careful consideration with an experienced genetics team.

## Interpreting the genomic test report

The clinical report should incorporate all the aspects of variant curation we have just described to state whether a pathogenic variant associated with the phenotype has been identified. When applicable, additional findings should also be noted. The ACMG guidelines, whose goals are to avoid false-positive or false-negative results to ensure accurate diagnoses, provide scores to help standardise this evaluation. The scores correspond to one of five variant classification assertions: class 5, pathogenic; class 4, likely pathogenic; class 3, variant of uncertain significance (VUS); class 2, likely benign; and class 1, benign.

Counselling patients as to the meaning of their results requires clinicians to have a solid understanding of their clinical implications and their potential impact on reproductive decisions. Only class 4 or 5 variants are clinically diagnostic. Any variant may be upgraded or downgraded with time as more data become available, provided the testing laboratory provides such a re-analysis service. The clinical report should clearly state the variant class and the data used to support this decision. Ideally, the nephrologist should have a thorough understanding of the meaning of the report and if not involved in its preparation should have the ability to challenge the result where it is not concordant with phenotype. The ACMG guidelines were designed for evaluating patients with a well-defined phenotype [17]. Thus, they should be used with caution for screening healthy individuals for whom we do not have a clear understanding of the impact of pathogenic variants and poorly phenotyped patients, such as those with CKD of unknown cause, where a lack of clear phenotype could mean downgrading a potential diagnostic variant. Expertise and support in interpreting reports or addressing any problems with the sequencing or interpretation process may be gained from clinical genetics colleagues and the laboratory itself depending on the available genomic medicine service provisions.

## Consideration of at-risk family members

Genomic testing, like other diagnostic modalities, can have important ramifications not only for the patient in front of you, the proband, but also for other family members. Cascade screening is particularly relevant for diseases in which early diagnosis could lead to pre-emptive treatment or screening measures, as in the case of X-linked Alport syndrome caused by *COL4A5* variants, where early treatment with an angiotensin-converting enzyme (ACE)-inhibitor can delay the progression of CKD [75]. This can further have implications for reproductive decision-making, in the forms of carrier or prenatal screening. In such cases, pre- and post-test counselling with a licensed genetic counsellor should be offered for all family members to support fully informed and autonomous decision-making [76].

## Indications for re-analysis of sequencing data

Existing sequencing data should be re-analysed where there is high clinical suspicion of a genetic cause but a negative test result, especially if there is a change in patient phenotype. With time, variants of uncertain significance may be upgraded or downgraded as new data are accrued. Furthermore, new gene-disease relationships published since the last analysis may identify causative genetic changes in previously unexamined genes. A time interval of between 12 and 24 months from initial test is recommended for variant re-analysis [77–79].

## Limitations of genomic sequencing

### Content of genomic test reports

Despite a common structure, the content of genomic sequencing reports can vary across providers. Nephrologists should seek to establish methods in which they can access support from their geneticist colleagues, genetic counsellors, testing laboratory, or bioinformaticians depending on their local set-up. This can enable them to access assistance as required.

### Challenging areas of the genome to sequence

The nephrologist should know if the gene they are focused upon is technically challenging to sequence as this can result in false-negative test results. Structural variants, highly repetitive sequences, multiple homologous elements, or extreme guanine-cytosine content are challenging to accurately map. Many challenges presented by trying to identify diagnostic variants in difficult to map regions can be offset by communication with the testing laboratory and clinician involvement in gene curation. Here, we highlight a few prominent examples in nephrology.

Short CNVs can be detected using ES (< 50 kb), but longer CNV can be difficult to detect [80]. Use of CMA or WGS can increase sensitivity of detecting CNV. The ability for CNV analysis to make molecular diagnoses is particularly substantial in children with CAKUT. In a cohort of 522 children with CAKUT who underwent CNV detection via CMA, 14.5% with isolated CAKUT had a pathogenic CNV (including deletions in *HNF1B*); this percentage increased to 22.5% if the child additionally had malformations outside of the kidney [81]. In total, 55/522 (10.5%) cases with CAKUT harbored 34 distinct known genomic disorders, which were detected in only 0.2% of 13,839 population controls. Another 32 (6.1%) of patients with CAKUT cases harbored large gene-disrupting CNVs that were absent from or extremely rare in the control group. The control group consisted of anonymised adults and children selected from six cohorts of European (80.4%), Asian (13.4%), and African American ancestry (6.1%). In an Australian study, 18 of 178 children with CAKUT were shown to have CNVs in genes known to be associated with kidney disease including *EYA1* [82]. Performing CMA prior to ES is one option to increase the efficiency and diagnostic yield. We suggest performing CMA for probands who, in addition to their kidney disease, also have intellectual disability, developmental delay, or other syndromic features as CNVs can be more prevalent in these cases [15]. For instance, the combination of autism spectrum disorder and insulin-dependent diabetes may suggest a 17q12 deletion, which encompasses the gene *HNF1B* and is associated with kidney cysts and diabetes syndrome. As CMA is also a genome-wide test, this strategy has been shown to identify incidental microdeletions or duplications and families should be counselled accordingly. Examples of this include the incidental finding of a cancer predisposition syndrome due to an *APC* gene deletion when investigating a patient with features of 22q11.2 syndrome (DiGeorge syndrome) leading to early surveillance for hepatoblastoma that was successfully detected and treated [83, 84]. Of note, different laboratories use different probes, so it is important to ask if there are sufficient probes in the region that harbor known kidney genes or kidney-related CNVs, such as *HNF1B.*

One limitation of ES is the ability to accurately sequence genes that have pseudogenes. A pseudogene is a section of DNA that structurally resembles a gene, but does not encode a protein, due to accumulated mutations that have occurred throughout evolution [48]. The *PKD1* gene, implicated in the development of ADPKD, has traditionally been difficult to accurately sequence via ES because it has 97% sequence similarity to six nearby pseudogenes [85]. It is important to recognise and consider this in a patient in whom you highly suspect ADPKD with an ES-negative result, which may be due to sequencing limitations. Discussion with clinical genetics may help to identify alternative sequencing strategies, such as Sanger sequencing of *PKD1*, to overcome this issue [39].

Besides repetitive sequences, other sequence features like variable number tandem repeats (VNTR) can also be difficult to capture through massively parallel sequencing techniques. The *MUC1* gene, responsible for autosomal dominant tubulointerstitial kidney disease (ADTKD), remains challenging for both ES and WGS because there is an insertion of a single C in one copy of the repeat unit of a long VNTR of the gene. However, single-molecule real-time (SMRT) sequencing has been used to successfully detect *MUC1* variants and is being introduced to the clinical setting [40].

Finally, false-negative results may occur if, for some technical reason, there is insufficient ‘coverage’ of a genomic region which prevents variants from being called. Coverage is a measure of how much of the genome has been sequenced at a particular depth, describing how often bases from the reads cover a sequence. The sequencing depth refers to the number of times a single base in the genome is sequenced. Therefore, when a clinician examines a negative clinical genetic test, they should assure themselves that the testing laboratory achieved sufficient coverage across the genomic regions of interest.

### Incidental findings

Incidental, or secondary findings, are genomic results that are not related to the indication for genomic testing but may be of medical utility [41]. One of the earliest recognised incidental findings were genetic parentage and parental consanguinity, which can have significant family and social implications. The risk of incidental findings increases as genomic testing becomes broader, being the highest for WGS.

The ACMG has curated a short list of actionable genes in which incidental findings must be reported [41]. An actionable gene is one within which a pathogenic or likely pathogenic variant will result in a disease, and for which there are specific evidence-based interventions or treatments that will improve morbidity and/or mortality risk. An example of this is finding pathogenic variants in the *BRCA1/BRCA2* genes which can cause hereditary breast and ovarian cancer and identification directly impacts therapeutic planning for the patient [41]. Pathogenic variants in genes deemed actionable should have a high penetrance. Outside of this list of actionable genes, incidental variants may be identified through broad genetic testing that warrant thoughtful consideration by the nephrologist (e.g. pathogenic ADPKD-associated variants) [65]. Unlike ES or WGS, there are no stringent guidelines for incidental CNVs identified by CMA, although such findings are not rare [84]. The risk of these different incidental findings underscores the importance of calibrating genomic testing to the clinical case and indication. For instance, CNV analysis is advised in severe clinical cases such as those with neurodevelopmental disorders, multiple congenital anomalies, and severe fetal anomalies which can include kidney anomalies [46]; on the other hand, the relevance of incidental CNVs in a mild clinical case is questionable. It is important, however, to recognise that the absence of incidental findings does not necessarily indicate that an individual is not at risk for a condition, but rather that no pathogenic variant based on our current scientific evidence was identified. Any provider in a kidney genetics program should be prepared to disclose incidental finding results, counsel on their implications, and arrange for appropriate referrals and follow-up.

### Negative results

A negative result from diagnostic genomic or genetic testing does not completely rule out a genetic disease. Reasons for a negative test may be that the relevant gene or area of the genome was not analysed or could not be captured using the test applied. Alternatively, there could be undiscovered causative variants in the genome analysed, which may become apparent once significant evidence base is published regarding their role. Therefore, a negative result in this context should be interpreted as no genetic cause was found around the genome analysed at that moment rather than the patient does not have the specific genetic disease. These patients can also have their results re-analysed if there is strong clinical suspicion of genetic disease despite a negative result. A negative result in the context of carrier testing is slightly different. As this test looks for one disease secondary to one specific genetic change, a negative result can rule out that specific genetic change as a cause of the familial disease.

## Establishing networks of support

Creating clear, easily accessed communication pathways between nephrologists and the genomic testing laboratory can help increase the diagnostic yield. Depending on local practice and legislation, these may take the form of multi-disciplinary meetings, messaging incorporated into the medical record or emails. This is particularly useful for information sharing, such as cases with an evolving phenotype or when a lab requires clinician input to help with variant interpretation. Nephrologists may also wish to clarify genomic test reports, especially when variants of uncertain significance have been found, to help identify how further evidence can be obtained to prove pathogenicity. These questions can also be directed to clinical genetics colleagues.

## Conclusion

With increasing evidence to support the clinical use of genomic testing in our patients and the improvement in cost and availability of genomic sequencing, it becomes more important for pediatric nephrologists to carefully consider if testing will benefit each patient they see. As with any other test, nephrologists should consider the reasons behind ordering a genomic test, the pre-test probability of a positive result, whether the test will provide a definitive molecular diagnosis, perhaps even altering a previous erroneous diagnosis, and whether there are meaningful management implications to gain from the test [19]. After deciding to perform sequencing, nephrologists should be aware of the intricacies of sequencing analysis and result interpretation to counsel a patient on test results appropriately. Like any other test, some of these intricacies include awareness of the chance of false-positive or false-negative results, equivocal results (‘variants of uncertain significance’), or incidental findings unrelated to the reason for testing but which could have substantial clinical impact on the patient and their relatives.

### Key summary points

- Whether to undertake a genetic or genomic test should involve considering the potential clinical benefit as well as diagnostic utility, which may outweigh the latter in certain indications.
- There are several different types of genomic test, such as panel, exome, whole-genome sequencing, and chromosomal microarray. The type of test pursued for an individual patient should be selected based on careful phenotyping, an understanding of the genetic heterogeneity of the suspected genetic disorder, and technical features of each test (e.g. gene coverage and sequencing depth).
- Familiarity with variant interpretation is key to understanding and explaining outcomes from a clinical report.
- Incidental findings which are unexpected and unrelated to the original reason for testing may have clinical significance. These results should be interpreted and reported based on the American College of Medical Genetics guidelines.

## Multiple-choice questions

Answers are provided following the reference list.

1. In which of these patients would a chromosomal microarray be most likely to yield a positive result?

- 10 year old with isolated SRNS
- 12 year old with CKD of unknown etiology
- 17 year old with cystic dysplasia and diabetes
- 8 year old with microscopic hematuria

2. ACMG guidelines for variant interpretation includes consideration of which of the following parameters:

- Allele frequency
- Segregation data
- In silico functional prediction tools
- All of the above

3. Clinical utility of genomic testing may include which of the following:

- Starting surveillance for extra-renal features
- Avoiding a kidney biopsy
- Modify pharmacological therapy
- All of the above

**Multiple choice answers:** 1, c; 2, d; 3, d.

## Supplementary information

Below is the link to the electronic supplementary material.

Graphical abstract (PPTX 453 KB)

## Data Availability

All data supporting the findings of this study are available within the paper and its supplementary information.
