# Supplementary material for: Next-generation nephrology: part 1—an aid for genetic and genomic testing in pediatric nephrology
Source: Pediatr Nephrol. 2025 Feb 13;40(9):2759–77. doi: 10.1007/s00467-025-06697-2 (PMC12297000; doi:10.1007/s00467-025-06697-2)
Supplement: Supplementary file 1 — Graphical abstract (PPTX 453 KB) [file 467_2025_6697_MOESM1_ESM.pptx]

## Slide 1
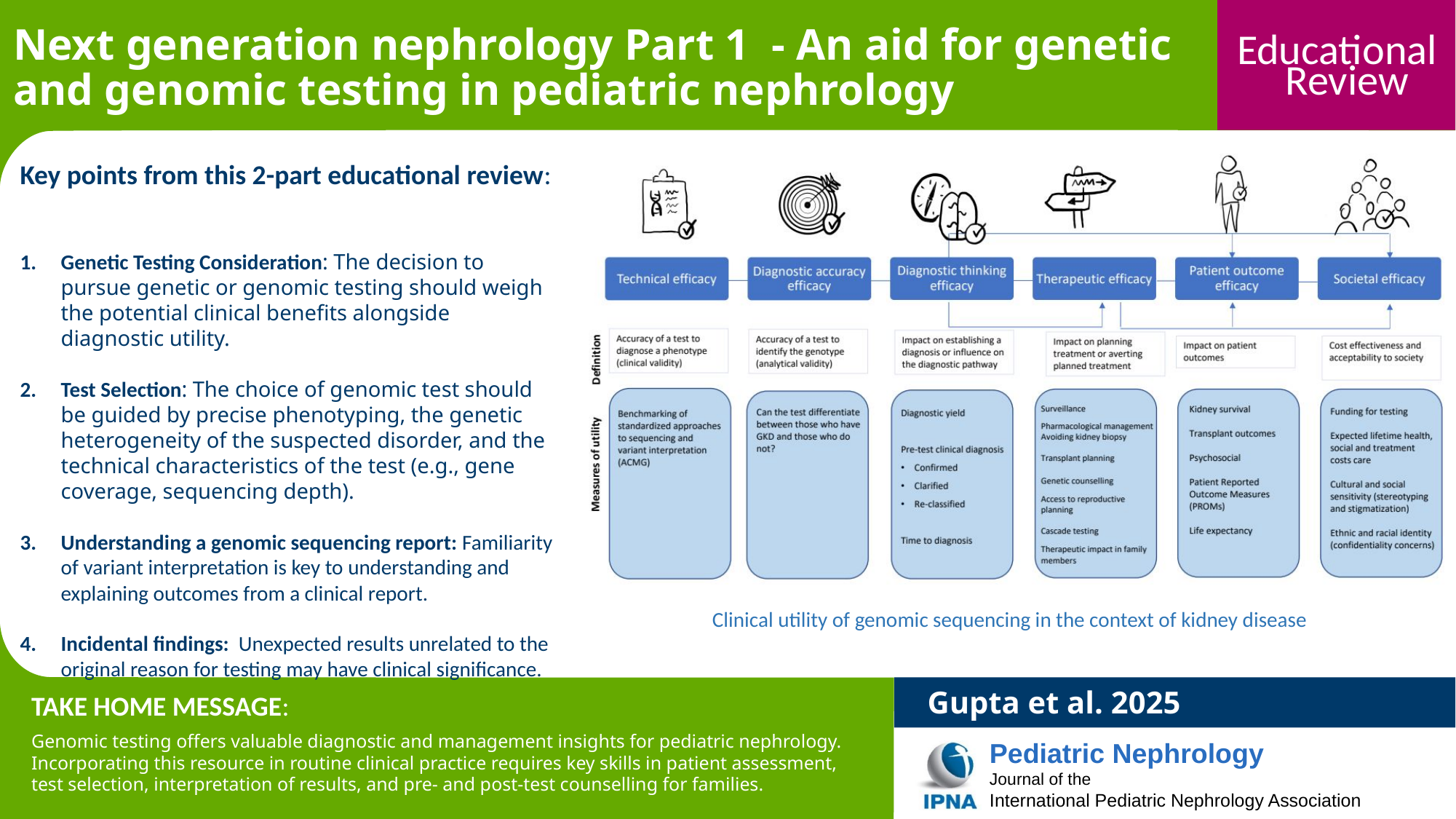

Next generation nephrology Part 1 - An aid for genetic and genomic testing in pediatric nephrology
Key points from this 2-part educational review:
Genetic Testing Consideration: The decision to pursue genetic or genomic testing should weigh the potential clinical benefits alongside diagnostic utility.
Test Selection: The choice of genomic test should be guided by precise phenotyping, the genetic heterogeneity of the suspected disorder, and the technical characteristics of the test (e.g., gene coverage, sequencing depth).
Understanding a genomic sequencing report: Familiarity of variant interpretation is key to understanding and explaining outcomes from a clinical report.
Incidental findings: Unexpected results unrelated to the original reason for testing may have clinical significance.
Clinical utility of genomic sequencing in the context of kidney disease
Gupta et al. 2025
TAKE HOME MESSAGE:
Genomic testing offers valuable diagnostic and management insights for pediatric nephrology. Incorporating this resource in routine clinical practice requires key skills in patient assessment, test selection, interpretation of results, and pre- and post-test counselling for families.
